# Human Papillomavirus (HPV) in breast tumors: prevalence in a group of Mexican patients

**DOI:** 10.1186/1471-2407-9-26

**Published:** 2009-01-22

**Authors:** David Cantu de León, Delia Pérez Montiel, Jana Nemcova, Iva Mykyskova, Elmer Turcios, Verónica Villavicencio, Lucely Cetina, Alberto Coronel, Ondraj Hes

**Affiliations:** 1 Department of Gynecologic Oncology, Instituto Nacional de Cancerología, México City, México; 2 Department of Pathology Instituto Nacional de Cancerología, México City, México; 3 Molecular Laboratory at Sikl's Department of Pathology Medical Faculty Hospital, Charles University, Plzen, Czech Republic; 4 Department of Surgical Oncology. Instituto Nacional de Cancerología, México City, México

## Abstract

**Background:**

Breast cancer is one of the main health problems in developed countries, occupying first place in mortality in women. It is well-known that there are risk factors associated with breast cancer development. Nonetheless, in 50–80% of cases known risk factors have not been identified, this has generated the attempt to identify new factors related with this neoplasia as viral infections. The aim of this work is investigate the prevalence of HPV DNA in patients with breast lesions at the Instituto Nacional de Cancerologia de Mexico.

**Methods:**

Fifty-one cases of breast cancer were selected from the files of the institute and compared by age and tumor size with 43 cases of non malignant breast lesions (fibroadenoma, fibrocystic disease and phyllodes tumor). Paraffin embedded specimens were selected, HPV DNA was analyzed by polymerase chain reaction (PCR) and sequenced for different types of HPV in case of positivity for HPV-DNA. Descriptive analysis of clinical and pathological variables was performed and comparisons between positive and negative cases was done.

**Results:**

All patients were mexican, mean age was 53.3, median age of menarche was 13 and median tumor size 9 cms. Cervicovaginal cytology was performed to all patients, 1 patient (1.9%) of cancer group had HPV and none in the other group, no cases were diagnosed with cervical dysplasia. In the group of carcinomas 36 (70.5%) were negative and 15 (29.4%) were positive to HPV-DNA, 10(66.6%) were positive for HPV 16, 3(20%) for HPV 18, two cases (13.4%) were positive for both. In the group of benign conditions all were negative to HPV-DNA.

**Conclusion:**

Presence of HPV in breast cancer in our group of cases is high in comparison to other authors; larger numbers of cases need to be analyzed in order to establish the exact role of this virus in the pathogenesis of breast cancer.

## Background

In developing countries, breast cancer occupies second place in frequency, preceded only by cervical cancer, as has been observed in reports on Mexico [1]. Worldwide, breast cancer is one of the main health problems in developed countries, occupying first place in mortality in women [2].

Breast cancer in Mexico continues to be one of the principal health problems for feminine population. In the year 2003, the Mexican General Epidemiology Directorate reported 12,433 (11.3%) new cases of invasive breast cancer and 517 (0.4%) cases of cancer *in situ*, presenting a mortality rate of 7.43/100,000 inhabitants [3].

It is well-known that there are risk factors associated with breast cancer development (age, familial history, personal history of breast cancer). Nonetheless, in 50–80% of cases known risk factors have not been identified, which has generated the attempt to identify new factors related with this neoplasia[1].

Recent studies suggest the association of viral infections with breast cancer pathogenesis, such as Epstein-Barr virus (EBV) [4] and mouse mammary tumor virus (MMTV) [5]. In addition, human papilloma virus (HPV) DNA sequences have been isolated. The relationship of HPV with other neoplasms in other anatomic sites (anogenital, upper aerodigestive tract, and skin) is well-known. Therefore, we know that HPV is related with 99.7% of cervicouterine carcinomas, and that there exist specific types that are associated with pre- and malignant cervical lesions [6].

Correlation of DNA identification of HPV and breast cancer ranges in variability from 0–86% of cases [7], and moreover, this has not been related with anogenital pathology. To date, the mechanism by which the virus reaches the breast has not been clearly identified [8].

At present, studies evaluating the presence of HPV in mammary lesions have practically all been conducted in Europe and Asia; we have identified two carried out in the U.S. [9,10] and one in Brazil [8].

We conducted this retrospective study with a representative number of malignant and non epithelial breast lesions in order to investigate the frequency of HPV in both types of tumors in a group of population different to the ones studied by others.

## Methods

### Patients and specimen collection

All patients were selected from the clinical archives at the Instituto Nacional de Cancerología de México. Cases selected from January 1999 to December 2003 in whom complete information from the chart was available were eligible for further evaluation.

From all cases evaluated in our institution 65 were chosen, slides and paraffin blocks located and reviewed by two pathologist, the diagnosis of breast cancer was confirmed. Most representative blocks were selected for HPV typing.

Clinical information was retrieved from the clinical file, such as: Age at diagnosis, age of menarche, family history of breast cancer, previous history of tobacco use, cancer stage, tumor size, histology, grade of tumor, Scarff-Bloom-Richardson (SBR) grading, estrogen receptors and progesterone receptors status. Forty three cases of non malignant breast lesions were used as controls (17 phyllodes tumors, 14 fibroadenoma and 12 fibrocystic disease).

DNA isolation and HPV typing were performed at the Molecular Laboratory at Sikl's Department of Pathology Medical Faculty Hospital, Charles University, Plzen, Czech Republic.

Data analysis was performed with SPSS 15.0 for windows (SPSS Inc, USA). Descriptive analysis of clinical and pathological variables was performed and comparisons between positive and negative cases were by chi-square test and student-*t *when appropriate. Statistical significance was accepted at the 5% level. To obtain a power of at least 80% a sample size was calculated and a minimum of 45 cases must be evaluated for comparisons. This protocol was evaluated and approved by the internal review board of both institutions.

### DNA isolation

Five 5-μm-thick sections were cut from FFPE tissue. After every sample, the knife was cleaned with xylene and ethanol. Negative controls consisted of extracted 5-μm-thick slices of paraffin blocks containing no tissue and cut in between the tissue samples. DNA was extracted by the DNeasy Tissue Kit (QIAGEN, Hilden, Germany) according to manufacturer's protocol. Quality of isolated DNA was checked by PCR of control genes with primers generating fragments of 100, 200, 300, 400, and 600 bp [11].

### HPV PCR systems

According to the quality of DNA different PCR systems for HPV detection and typing were used. If there was possible to amplify more than 400 bp fragment of control genes from DNA of sample (27 samples), nested PCR using MY09/11 and GP5+/6+ was performed. Degenerated primers MY09 (5'CGTCCMARRGGAWACTGATC 3') and MY11 (5'GCMCAGGGWCATAAYAATGG 3') [12] amplify 450 bp long fragment in highly conserved region in L1 gene. Consensus primers GP5+ (5'-TTTGTTACTGTGGTAGATACTAC-3') and GP6+ (5'-CTTATACTAAATGTCAAATAAAAA-3') [13] generate 140 to 150 bp fragment of the L1 region of the virus. Both systems of primers detect a broad spectrum of oncogenic and nononcogenic mucosal and some mucocutaneous HPV types.

In the case of samples, where control genes were amplifiable in range 100–200 bp only (14 samples), the one-step PCR with primers GP5+/6+was used. In samples of even worse quality of DNA (10 Samples) an INNO-LiPA HPV Genotyping kit (Innogenetics NV, Ghent, Belgium) were used. The INNO-LiPA HPV Genotyping kit permits ultrasensitive detection of a broad spectrum of HPV genotypes by PCR with primers SPF10 that amplifies a 65 bp long region in the HPV L1 gene, and typing by a reverse hybridization (LiPA) [14].

### HPV detection

Reaction mixture (50 μl) for nested PCR consisted of 1× reaction buffer (Promega), 4 mM MgCl_2_, dNTPs 0.2 mM of each, primers MY09/11 0.5 pmol each, 2.5 U Taq polymerase (Promega) and 5 μl of isolated DNA. 3 μl of PCR product from first step were added into reaction mixture with GP5+/6+ primers, which had identical composition as in first step except for 3.5 mM MgCl_2_. PCRs were run using folloving profile: initial denaturation at 95°C for 5 min, 40 cycles of 95°C for 1 min, either 50°C (annealing for MY09/11 primers) or 40°C (GP5+/6+ primers) for 1 min and 72°C for 1 min, final incubation by 72°C for 5 min. In each reaction positive control (HPV positive clinical sample from cervical smear, in which the HPV 16 was confirmed by sequencing) and negative control were included. Negative controls were never found to be positive. PCRs were run on the cycler GeneAmp PCR system 2400 (PE/Applied Biosystems, Foster City, CA). Products of all PCRs were separated in a 2% agarose gel (Agarose for DNA electrophoresis, Serva, Heidelberg, Germany). Successfully amplified PCR products were purified with a QIAquick spin PCR purification kit (QIAGEN), sequenced using a Big Dye Terminator Sequencing kit (PE/Applied Biosystems), and run on an automated sequencer ABI Prism 310 (PE/Applied Biosystems) at a constant voltage of 11.3 kV for 20 minutes. Results were evaluated by BLAST program http://www.ncbi.nlm.nih.gov/BLAST.

Inno-LiPA genotyping was performed according to manufacturer's protocol.

## Results

Of 65 patients selected per group of breast cancer, 51 specimens showed DNA integrity by control genes amplification, mean age was 53.3 ± 13.2 years (range 27–82), age of menarche 12.9 ± 1.4 years (range 10–16), 66% (42) of patients did not have family history of breast cancer, of the patients with family history of breast cancer 12 (18%) had at least one family member with the disease, only 16.9% of patients (11) were smokers. Cervicovaginal cytology was performed to all patients, 1 patient (1.9%) of cancer group had HPV and none in the other group, no cases were diagnosed with cervical dysplasia.

Forty-eight tumors (94.1%) were ductal carcinomas, tumor size ranged 1–17 cms (mean 9.4 cms), 11 tumors (16.9%) were SBR 8 and 10 tumors (15.4%) were SBR 9. Estrogen receptors were positive in 15 cases (23%) and progesterone receptors were positive in 16 (24.6%).

**Figure 1:**
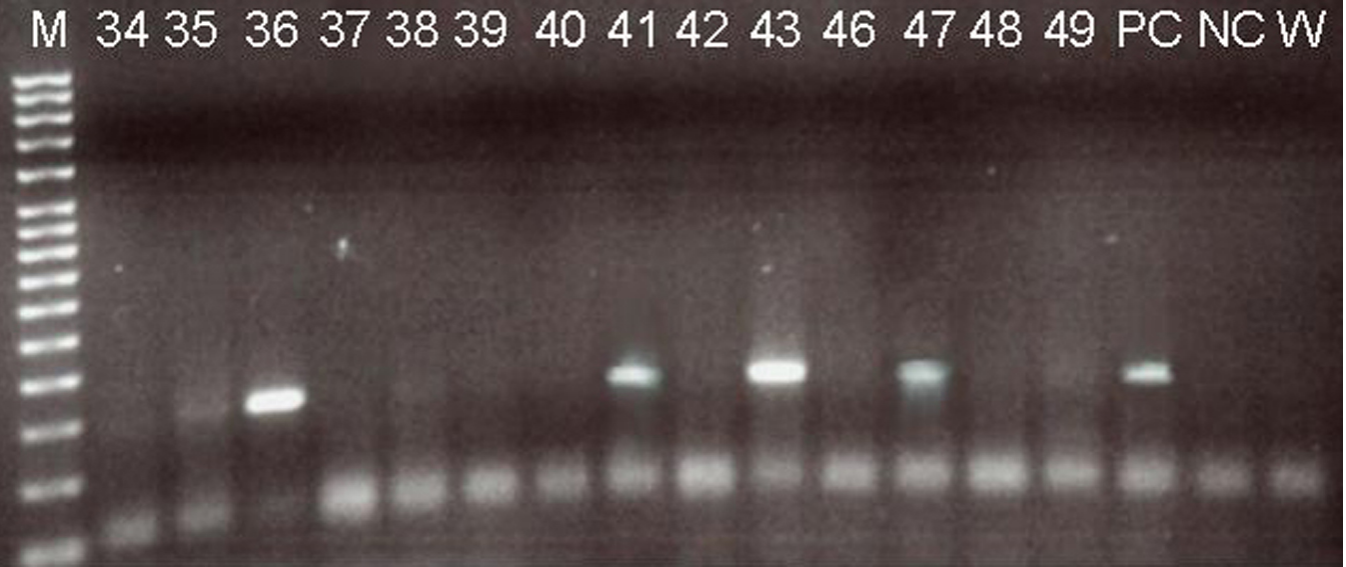
**Electroforetical analysis of nested PCR products**. In left line – MW marker, lines marked 34 – 49 are negative and positive samples, PCR product size 150 bp, **+ **– positive control (described in protocol), **0,0 **– double negative control (PCR premix with no DNA).

Fifteen cases were HPV positive (29.4%) and 36 cases (70.5%) were negative, when typing was performed, 10 (66.6%) were positive for HPV 16, 3 (20%) for HPV 18, and two cases (13.3%) were positive for HPV 16 and 18. (Fig 1)

When comparisons between HPV positive and negative were done in relation to all clinical and pathological variables the only one which was significant was tumor size, since only one tumor larger than 4 cms was positive for HPV (p = 0.008), as shown in Table 1.

**Table 1:** Comparisons of HPV status and clinical variables.

	HPV (+) n = 15	HPV (-) n = 36	P value
Age	48.13 ± 12.35	55.36 ± 13.5	0.86

Histology			0.489

Ductal	15	33	

Lobular	0	3	

Clinical stage			0.206

Early	5	7	

Advance	7	26	

*Not classificated*	3	3	

Estrogen receptors			0.342

Negative	7	13	

Positive	5	8	

Not done	3	15	

Progesterone receptors			0.190

Negative	6	15	

Positive	6	6	

Not done	3	15	

Tumor size			**0.008**

**< 2 cm**.	**5 (33.33%)**	**5 (13.88%)**	

**2 – 4 cm**.	**9 (60.00%)**	**12 (33.33%)**	

**> 4 cm**.	**1 (6.66%)**	**19 (52.77%)**	

All non-malignant breast lesions were negative for HPV.

## Discussion

The majority of molecular events in the genesis of breast cancer are unknown. However, initial studies have reported an association of breast cancer with cervical intraepithelial neoplasia III (CIN III)-like lesions [9]. These studies have aroused interest in the search for HPV as part of breast cancer genesis.

In 1992, Di Lonardo and colleagues were the first to demonstrate the association of HPV in 29.4% of 17 patients with breast cancer, identifying HPV 16 DNA by means of PCR [15]. In 1999, Yu et al. in a study group of 72 patients published on the association between HPV 33 and breast cancer in an Oriental population (China and Japan), suggesting the presence of HPV 33 DNA in invasive ductal carcinoma (IDC) in 34.1% of patients studied and in 5% of benign lesions, orienting thought toward its participation in the pathogenesis of breast cancer, but not for other serotypes [16].

Hennig et al. reported the association of HPV 16 in 19 of 41 breast carcinomas (46%) in patients with a history of CIN III lesions. HPV 16 DNA was detected in cervical lesion in 32 of 38 patients with CIN III (84%). All patients with HPV 16 positive for breast cancer corresponded to the same patients with HPV 16 in CIN III lesions; there were no cases with HPV 11, 18, or 33. HPV 16 was detected in primary breast tumors, as well as in lymph node metastases, in addition to a case of distant breast cancer metastasis with HPV 16 to colon. There was no correlation with the histology of breast tumor, tumor size, or lymphatic affectation, but a slight association was demonstrated between p53 and -21 expression in patients with breast cancer and HPV 16 [17]. Hormonal receptors were quantified, but no statistically significant difference was found [18], this finding is similar to ours.

Yu in 2000 again published the correlation of HPV DNA in 14 of 32 (43.8%) cases of patients with intraductal carcinoma, finding HPV 33 (but not HPV 16 or 18) DNA in the positive cases. They suggested that HPV 33-associated infection had a place in the pathogenesis of breast cancer in the study population [19].

Liu et al. reported a considerable number of HPV-related mammary lesions. They identified protein E6 and -7 in six of 17 (35%) cases of patients with breast cancer. Cloned HPV sequences identified DNA in serotypes 16, 18, and probably 11, concluding that the same changes in p53 and -21 in cervix can be produced in breast, thus suggesting the mechanism by means of which HPV can exert an influence on the genesis of breast cancer [9].

Damin and colleagues detected HPV in 25 of 101 (24.75%) patients with breast cancer, and did not identify HPV in any of the 41 benign lesions studied (20 patients reduction mammoplasty and 21 with mammary fibroadenomas). In 14 (56%) patients, the authors isolated HPV 16 DNA, in 10 (40%) patients HPV 18, and in one patient (4%) both serotypes, this study with the highest case number [8].

Our results are similar to those reported in the world literature, in which a prevalence of between 24 and 46% is reported in the association of invasive breast cancer and HPV-related infection [8,9,15-20]. In our study group, we identified HPV-positive tumors in 29.4% of cases included in the study. HPV 16 DNA was most frequently found (66.6%), as reported by other studies [9,15,17-20], in which Western populations were included; the DNA of other serotypes was not identified in this study group. We are aware of some possible discrepancies between analytical sensitivity of the used PCR approaches even with the special precautions to avoid minimal contamination that we had; these possible discrepancies between the two techniques might be the main limitation of our results. We found no viral DNA presence in any benign lesions and in any phyllodes tumors included in our series.

Other authors found no association between HPV presence and breast cancer, as is the case of Lindel [21], who found no viral DNA in a group of 81 cases of breast cancer.

In our series, tumor size was the sole statistically significant value, showing that the greater the lesion size, the greater the probability of finding viral DNA in the tumor sample but not larger than 4 cms where viral DNA seems to be lost or could not be apprised or retrieved. Nevertheless, this was no able to be correlated with histologic grade or with any poor prognosis factor, which is similar to the results of Kan et al. [16]; in these, while 48% were positive for HPV 18 a correlation was unable to be demonstrated with other pathologic variables or with patient outcome.

As reported by other authors for cancer – and even breast cancer – at other localizations, expression of protein E6 and -7 was related with changes in p53 and -21, producing apoptosis inhibition, thus favoring cellular proliferation as part of breast cancer pathogenesis [17]. Although the mechanism by which HPV reaches the mammary gland is not clear, the possibility has been suggested by some authors of a viremia, explaining HPV installation in other organs including breast [20].

Notwithstanding this, it is important to consider the possibility of other epidemiologic factors, such as the geographic site from which cases were taken and HPV infection prevalence, to be able to determine HPV distribution in malignant breast lesions.

## Conclusion

The origin of breast cancer is multifactorial. The fact that we have not found viral DNA in benign lesions supports to a certain extent the etiologic role of the virus in at least a sub-population of patients. The mechanism involved is not clear. Thus, it is necessary to include a greater number of cases to determine the true role of the virus in the genesis of these lesions and in Mexican population were this neoplasm is a health problem research in this area is very important, therefore, a study like this, opens another possible explanation for the development of the disease.

## Competing interests

The authors declare that they have no competing interests.

## Authors' contributions

DC conceived of the study and participated in its design and statistical analysis. DPM participated in its design, collected and selection of the material and drafted the manuscript. JN carried out DNA isolation and HVP typing, drafted the manuscript. IM carried out DNA isolation and HPV typing, drafted the manuscript. EL participated in its design and drafted the manuscript. VV performed the statistical analysis. LC collected and selection of material, give clinical follow up. AC gave clinical follow up and critical review of the manuscript OH selection of material and drafted the manuscript.

## Pre-publication history

The pre-publication history for this paper can be accessed here:

http://www.biomedcentral.com/1471-2407/9/26/prepub
